# Identification of Multi-Functional Enzyme with Multi-Label Classifier

**DOI:** 10.1371/journal.pone.0153503

**Published:** 2016-04-14

**Authors:** Yuxin Che, Ying Ju, Ping Xuan, Ren Long, Fei Xing

**Affiliations:** 1 School of Information Science and Technology, Xiamen University, Xiamen, Fujian 361005, China; 2 School of Computer Science and Technology, Heilongjiang University, Harbin 150080, China; 3 School of Computer Science and Technology, Harbin Institute of Technology Shenzhen Graduate School, Shenzhen, Guangdong 518055, China; 4 School of Aerospace Engineering, Xiamen University, Xiamen, Fujian 361005, China; Harbin Institute of Technology Shenzhen Graduate School, CHINA

## Abstract

Enzymes are important and effective biological catalyst proteins participating in almost all active cell processes. Identification of multi-functional enzymes is essential in understanding the function of enzymes. Machine learning methods perform better in protein structure and function prediction than traditional biological wet experiments. Thus, in this study, we explore an efficient and effective machine learning method to categorize enzymes according to their function. Multi-functional enzymes are predicted with a special machine learning strategy, namely, multi-label classifier. Sequence features are extracted from a position-specific scoring matrix with autocross-covariance transformation. Experiment results show that the proposed method obtains an accuracy rate of 94.1% in classifying six main functional classes through five cross-validation tests and outperforms state-of-the-art methods. In addition, 91.25% accuracy is achieved in multi-functional enzyme prediction, which is often ignored in other enzyme function prediction studies. The online prediction server and datasets can be accessed from the link http://server.malab.cn/MEC/.

## Introduction

Enzymes play a crucial role in the catalysis of biological and chemical reactions. As effective catalyzers, they are not consumed and do not participate in the reactions. After they are catalyzed, more than 400 types of reactions can be accelerated. The enzyme commission (EC) number, which is based on the chemical reactions catalyzed by enzymes, is utilized to characterize different enzymes as a numerical classification scheme[1]. Enzymes are divided into six main classes, namely, oxidoreductases, transferases, hydrolases, lyases, isomerases, and ligases, and then subdivided into three hierarchical levels. Most studies on enzyme classification focused on monofunctional enzyme prediction. However, identification of the multifunctional enzyme, which is a specific type of enzyme that can catalyze two or more chemical reactions, has not been provided much attention.

Various approaches have been utilized to achieve high accuracy in monofunctional enzyme prediction. Bioinformatics approach has attained considerable achievements by using information on the protein sequence and structure[2]. Huang et al.[3] proposed an adaptive fuzzy k-nearest neighbor method with Am-Pse-AAC feature extraction method, which was first developed by Kou-Chen Chou for enzyme subfamily class prediction, and attained an excellent accuracy of 92.1% for the six main families. EzyPred[4] is a three-layer predictor that is based on PSSM; it considers protein evolutionary information abundant in the profiles. The second layer responsible for predicting the main function class achieves 93.7% accuracy. EFICAz[5] has a high accuracy of 92% in predicting four EC digit levels in a jackknife test on test sequences that are <40% identical to any sequences in the training dataset.

With regard to multifunctional enzyme prediction, Luna De Ferrari et al.[6] and Zou[7] achieved good results. Luna De Ferrari presented EnzyML, a multi-label classification method that employs InterPro signatures. This method can efficiently provide an explanation for proteins with multiple enzymatic functions and achieves over 98% subset accuracy without utilizing any feature extraction algorithms. Zou proposed two feature algorithms to make predictions and obtained 99.54% and 98.73% accuracy by using 20-D and 188-D features, respectively; however, dataset redundancy was not mentioned in the paper.

The enzyme sequence in the present study was obtained from the Swiss-Prot Database (release 2014.9), an authoritative organization that provides high-quality annotated protein sequences. After redundancy removal with cluster database—high identity with tolerance (CD—HIT)[8], the similarity of the sequence is established below 65% to ensure the effectiveness of the experiments. ACC is then applied[9, 10] for feature extraction. This method was first proposed by Dong as a taxonomy-based protein fold recognition approach and has not been utilized in enzyme classification yet. Accuracy of 94.1% in monofunctional enzyme classification is obtained by using the K-nearest neighbor classifier. With regard to multifunctional enzymes, an average precision of 95.54% and 91.25% is obtained after five cross-validation tests on all enzymes and multifunctional enzymes, respectively.

## Method

### Data preprocessing

The original downloaded dataset consists of 214,375 sequences. However, each enzyme class has duplicate sequences. 207,430 sequences remained after duplicate elimination. To eliminate the negative effect of sequence similarity, CD-HIT, a widely utilized procedure to reduce sequence redundancy and improve the performance of other sequence analyses using clustering (known as high computing speed) was applied to perform redundancy removal in the experiments. A total of 59,763 sequences with similarity below 65% were obtained. The CD-HIT algorithm progresses as follows. First, the http://cn.bing.com/dict/clientsearch?mkt=zh-CN&setLang=zh&form=BDVEHC&ClientVer=BDDTV3.5.0.4311&q=%E9%80%92%E5%87%8F%E6%8E%92%E5%BA%8F sequences are sorted in length-descending order. Second, the first series class is formed from the longest sequence, and subsequent sequences are compared with the representative sequence of the known series class. If the similarity is above the threshold set beforehand, the sequence is added in this class; otherwise, a new series class is formed. Third, the longest sequence is extracted from each class to form the final dataset. In the experiments, the threshold is set to 0.65, and the word length to compare is 5. Table 1 shows the situation before and after redundancy removal.

**Table 1:** Distribution of six enzyme classes before and after CD-HIT(0.65).

Dataset	EC 1	EC 2	EC 3	EC 4	EC 5	EC 6	Total
original data	32958	82735	38611	22754	14096	23221	214375
after duplicate-elimination	32016	79144	36862	22421	13872	23115	207430
after CD-HIT	8781	23716	11994	5331	4037	5904	59763

Notably, the multifunctional enzymes in the six classes have not been removed yet. Table 2 shows the distribution of multifunctional enzymes in the six classes.

**Table 2:** Distribution of multifunctional enzymes before and after CD-HIT(0.65).

Multifunctional enzymes	EC 1	EC 2	EC 3	EC 4	EC 5	EC 6	Total
before redundancy	1534	1924	2657	1698	616	179	4076
after CD-HIT	386	503	689	473	137	52	1085

### Feature extraction algorithm

#### Position-specific scoring matrix

For convenience of discussion, we denote a protein sequence as *S*, which is expressed as

$$S=s_{1}s_{2}s_{3}s_{4}\ldots s_{L},$$ (1)

where *L* represents the length of *S* and *s*_*i*_(1 ≤ *i* ≤ *L*) represents one item of the amino acid alphabet, which is expressed as {A, C, D, E, F, G, H, I, K, L, M, N, P, Q, R, S, T, V, W, Y}[11]. For sequence *S*, the position-specific scoring matrix (PSSM) was generated by implementing the PSI-BLAST program[12]. PSSM is a L*20 matrix[13] and can be expressed as follows:

$$PSSM={\left[\begin{array}{cccc} p_{1,1} & p_{1,2} & \cdots & p_{1,20}\\ \;p_{2,1} & p_{2,2} & \cdots & p_{2,20}\\ \vdots & \vdots & \vdots & \vdots \\ p_{i,1} & p_{i,2} & \cdots & p_{i,20}\\ \vdots & \vdots & \vdots & \vdots \\ p_{L,1} & p_{L,2} & \cdots & p_{L,20} \end{array}\right]}_{L\times 20}$$ (2)

where each row represents the corresponding position of *S* (e.g., the 1st row refers to *s*_1_, the 2nd row refers to *s*_2_, and so forth). Each column represents the corresponding residue type of the amino acid alphabet (e.g., the 1st column refers to “A,” the 2nd row refers to “C,” and so forth). *p*_*i*,*j*_(1 ≤ *i* ≤ *L*, *j* = 1,2,…, 20) is a score that represents the odds of *s*_*i*_ being mutated to residue type *j* during evolutionary processes; for example, *p*_1,1_ represents the odds of *s*_1_ being mutated to residue type “A”. A high score for *p*_*i*,*j*_ usually indicates that the mutation occurs frequently and that the corresponding residue in that position may be functional.

#### ACC feature representation algorithm

The framework consists of two feature models denoted as AC and CC. By using the PSSM of Eq (2), the enzyme sequence is formulated into a 20-D feature vector. The 20-D feature vector is calculated as

$$F\left(\bar{P_{j}}\right)=\left\{\bar{P_{j}}=\frac{\sum_{i=1}^{L}p_{i,j}}{L}|1\leq i\leq L;\;j=1,2,\ldots ,\;20\right\},$$ (3)

where $\bar{P_{j}}$ represents the average score of the amino acids in the enzyme sequence, which indicates the general odds of the sequence being muted to residue *j* during the evolutionary process.

In the model of AC, the enzyme sequence is computed as

$$F_{AC}=\{\frac{\sum_{i=1}^{L-\lambda }(p_{i,j}-\bar{P_{j}})*\left(p_{i+\lambda ,j}-\bar{P_{j}}\right)}{L-\lambda }|\;j=1,2,\ldots ,\;20\}.$$ (4)

As shown in Eq (4), *F*_*AC*_ measures the average correlation between two amino acids separated by a distance of *λ* in the enzyme sequence. The dimension of the feature vector *F*_*AC*_ is *λ* * 20.

In the model of CC, the enzyme sequence is computed as

$$F_{CC}=\{\frac{\sum_{i=1}^{L-\lambda }(p_{i,j_{1}}-\bar{P_{j}})*\left(p_{i+\lambda ,j_{2}}-\bar{P_{j}}\right)}{L-\lambda }|j_{1},j_{2}=1,2,\ldots ,\;20;\;j_{1}\neq j_{2}\}.$$ (5)

As shown in Eq (5), *F*_*CC*_ measures the average correlation between two amino acids separated by a distance of *λ* in the enzyme sequence among 20 types of standard amino acids. The dimension of the feature vector *F*_*CC*_ is *λ* * 380.

Combining *F*_*AC*_ and *F*_*CC*_ generates a (400 * *λ*)−*D* feature vector to represent the enzyme sequence, as represented by

$$F_{ACC}=\left(F_{AC}\;,\;F_{CC}\right).$$ (6)

The ACC feature representation algorithm fully employs the influence of the position correlation among sequence amino acids on protein homology detection. Secondary structure features[14, 15] were considered in other protein classification works. However, it is too time consuming for constructing web server.

### Classifier selection and tools

#### KNN algorithm

The K-nearest neighbors (KNN) algorithm is a mature method and is one of the simplest machine learning algorithms in theory. It is widely used for classification and regression. The key idea in this algorithm is that an object can be assigned to a class if the majority of its k nearest neighbors belong to this class. If k equals 1, then the object is simply assigned to the class of that single nearest neighbor.

**Fig 1:**
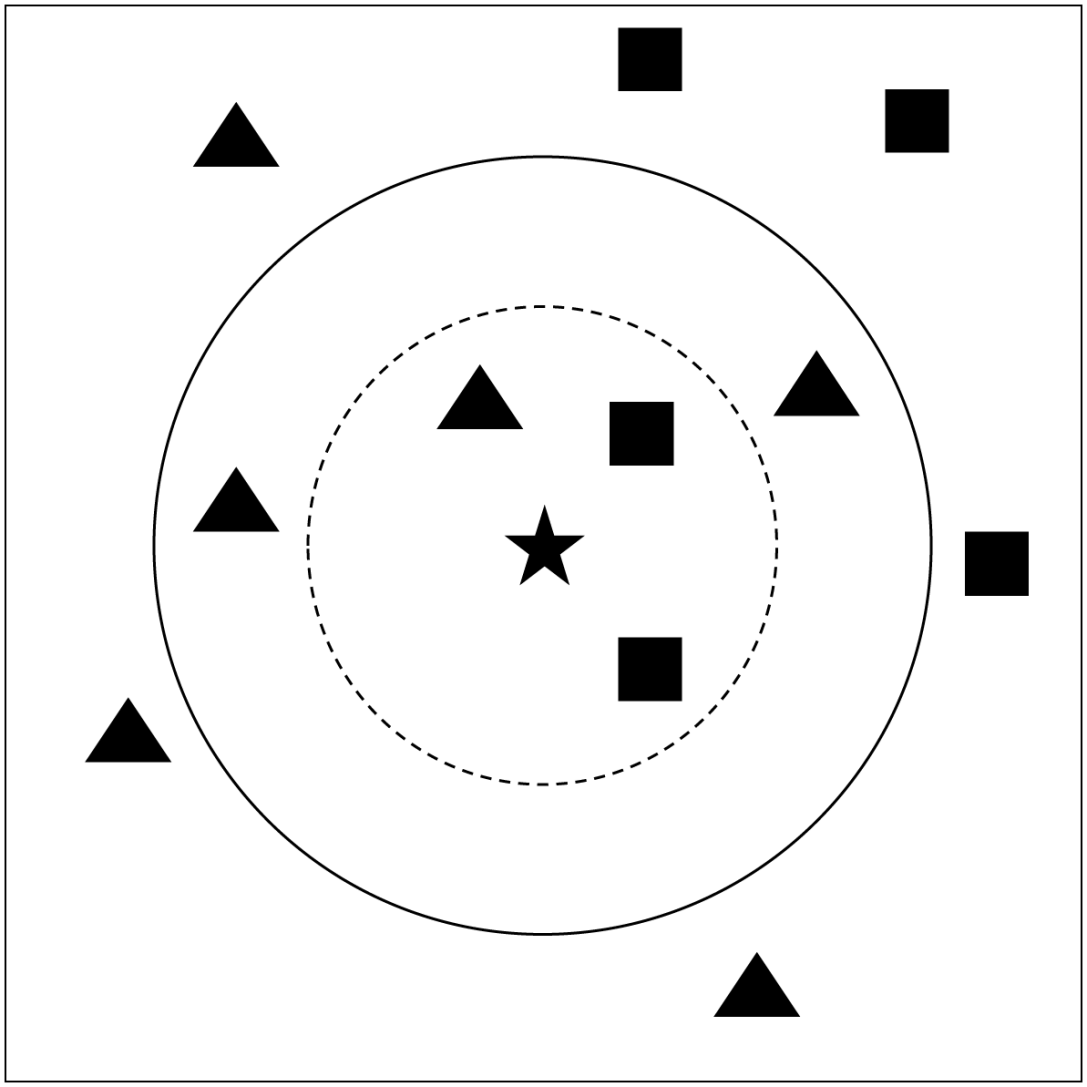
KNN algorithm diagram.

For instance, in Fig 1, the objective is to classify the test sample (star) either to the first class of triangles or to the second class of squares. If k equals three, we assign it to the second class according to dashed line circle because two squares and only one triangle exist inside the circle. If k equals five, we assign it to the first class according to the solid line circle because three triangles and only two squares exist inside the circle.

The choice of parameter k in this algorithm is important and depends on the data mostly. Generally, a large value of k dilutes the effect of noise in the classification but renders the boundaries between the categories less distinct. In our experiments, a large k value does not perform well.

KNN has been extensively utilized for the classification task in bioinformatics. Many recent studies have proven its high efficiency. In our experiments, we implemented a host of underlying classification algorithms and found that KNN is 20% more accurate than others.

#### WEKA and MULAN

Two of the main tools we utilized are Waikato environment for knowledge analysis (WEKA) and multi-label learning (MULAN). WEKA is an ensemble Java package with numerous machine learning algorithms and a graphical user interface. Several standard data mining tasks, including data preprocessing, feature selection, clustering, classification, regression, and visualization, are supported. MULAN is a Java library for learning from multi-label data. WEKA and MULAN contain an evaluation framework that calculates a rich variety of performance measures. They provide a convenient means to compare performance on different data using different classifiers.

### Measurement

#### Single-label measurement

Given multi-label test datasets *S* = {(*x*_*i*_,*y*_*i*_)|1≤ *i* ≤ *n*}, for class *y*_*i*_ where 1≤ *j* ≤ *m*, the binary classification performance of a predictor is presented by the four variables below.

$$TP_{j}=\left|\left\{x_{i}|y_{j}\in Y_{i}\wedge y_{j}\in h\left(x_{i}\right),\left(x_{i},Y_{i}\right)\in S\right\}\right|$$

$$FP_{j}=\left|\left\{x_{i}|y_{j}\notin Y_{i}\wedge y_{j}\in h\left(x_{i}\right),\left(x_{i},Y_{i}\right)\in S\right\}\right|$$

$$TN_{j}=\left|\left\{x_{i}|y_{j}\notin Y_{i}\wedge y_{j}\notin h\left(x_{i}\right),\left(x_{i},Y_{i}\right)\in S\right\}\right|$$

$$FN_{j}=\left|\left\{x_{i}|y_{j}\in Y_{i}\wedge y_{j}\notin h\left(x_{i}\right),\left(x_{i},Y_{i}\right)\in S\right\}\right|$$

*TP*_*j*_ indicates the number of true positive instances, *FP*_*j*_ indicates the number of false positive instances, *TN*_*j*_ indicates the number of true negative instances, and *FN*_*j*_ indicates the number of false negative instances. h(*x*_*i*_) indicates the classification results of sample *x*_*i*_ predicted by classifier h.

We obtained four evaluation performance indicators according to these four variables as shown below[1, 16–22].

$$\text{Accuracy}=\text{B}\left(TP_{j},FP_{j},TN_{j},FN_{j}\right)=\frac{TP_{j}+TN_{j}}{TP_{j}+FP_{j}+TN_{j}+FN_{j}}$$ (7)

$$\text{Precision}=\text{ B}\left(TP_{j},FP_{j},TN_{j},FN_{j}\right)=\frac{TP_{j}}{TP_{j}+FP_{j}}$$ (8)

$$\text{Recall}=\text{B}\left(TP_{j},FP_{j},TN_{j},FN_{j}\right)=\frac{TP_{j}}{TP_{j}+FN_{j}}$$ (9)

$$\text{F}-\text{measure}=\frac{2*Precision*Recall}{Precision+Recall}$$ (10)

#### Multi-label measurement

We employed two evaluation indicators[23], namely, example-based and label-based metrics. For example-based metrics, we calculated the classification results for each sample first and then obtained the average value for the entire dataset.

We considered multi-label classifier h and multi-label dataset S = {(*x*_*i*_,*Y*_*i*_)|1≤ *i* ≤ *n*}, where *Y*_*i*_ is the label collection of sample *x*_*i*_. *Y*_*i*_ = {0,1,1,0,1,0} denotes that sample *x*_*i*_ belongs to classes 1, 2, and 4 simultaneously.

$$Average\_precision_{s}\left(h\right)=\frac{1}{n}\sum_{i=1}^{n}\frac{1}{\left|Y_{i}\right|}\sum_{y\in Y_{i}}\frac{\left|y^{\prime }|rank_{f}\left(x_{i},y^{\prime }\right)\leq rank_{f}\left(x_{i},y\right),y^{\prime }\in Y_{i}\right|}{rank_{f}\left(x_{i},y\right)}$$ (11)

This index indicates the performance of the relevance tag emerging before a certain tag in the sorted class label sequences. The higher average precision is, the better the performance is; the best value is 1.

For label-based metrics, we calculated the binary classification results for each class first and then obtained the average value for all classes.

Based on single-label measurement, we supposed that B(*TP*_*i*_, *FP*_*i*_, *TN*_*i*_, *FN*_*i*_) represents the binary classification indicator. The following are defined.

$$B_{macro\;}=\frac{1}{m}\sum_{j\;=\;1}^{q}\text{B}\left(TP_{j},FP_{j},TN_{j},FN_{j}\right)$$ (12)

$$B_{micro}=\text{B}\left(\sum_{j\;=\;1}^{q}TP_{j},\sum_{j\;=\;1}^{q}FP_{j},\sum_{j\;=\;1}^{q}TN_{j},\sum_{j\;=\;1}^{q}FN_{j}\right)$$ (13)

*B*_*macro*_ measures the classification capability in each class and obtains the average of all classes as the final result. Its main idea is that each class shares the same weight. However, *B*_*micro*_ endows each sample the same weight. It calculates the sum of values in all classes and then utilizes the value to obtain classification capability as the final result. Such is the difference between these two indicators.

#### Multi-label classification ensemble algorithm

Suppose that m classifiers solve an n-class classification problem. We define score matrix scoreVectors, and scoreVectors(i,j) indicates the possibility of the sample being classified into class j by classifier i, where 0≤scoreVectors(i,j)≤1, 1≤i≤n, 1≤j≤m.

Similarly, we define binary matrix bipartitionVectors, and bipartitionVectors(i,j) represents whether the sample is classified into class j by classifier i, where bipartitionVectors(i,j)∈{0,1}, 1≤i≤n, 1≤j≤m.

Below are three ensemble methods.

$${\text{Mean}}_{\text{scoreVector}\left(\text{j}\right)}=\frac{\sum_{i=1}^{m}\text{scoreVectors}\left(\text{i},\text{j}\right)}{m},$$ (14)

$$\text{Majority}\_\text{bipartitionVector}\left(\text{j}\right)=\;\sum_{i=1}^{m}bipartitionVectors\left(i,j\right)\geq 0:1\;0,$$ (15)

$$\text{TopK\_scoreVector (j)}=\frac{\sum_{i=1}^{K}Sort\left(scoreVectors\left(i,j\right)\right)}{K},$$ (16)

where Sort(scoreVectors(i,j)) represents the scores being sorting in descending order.

## Result and Discussion

### Monofunctional enzyme classification

**Fig 2:**
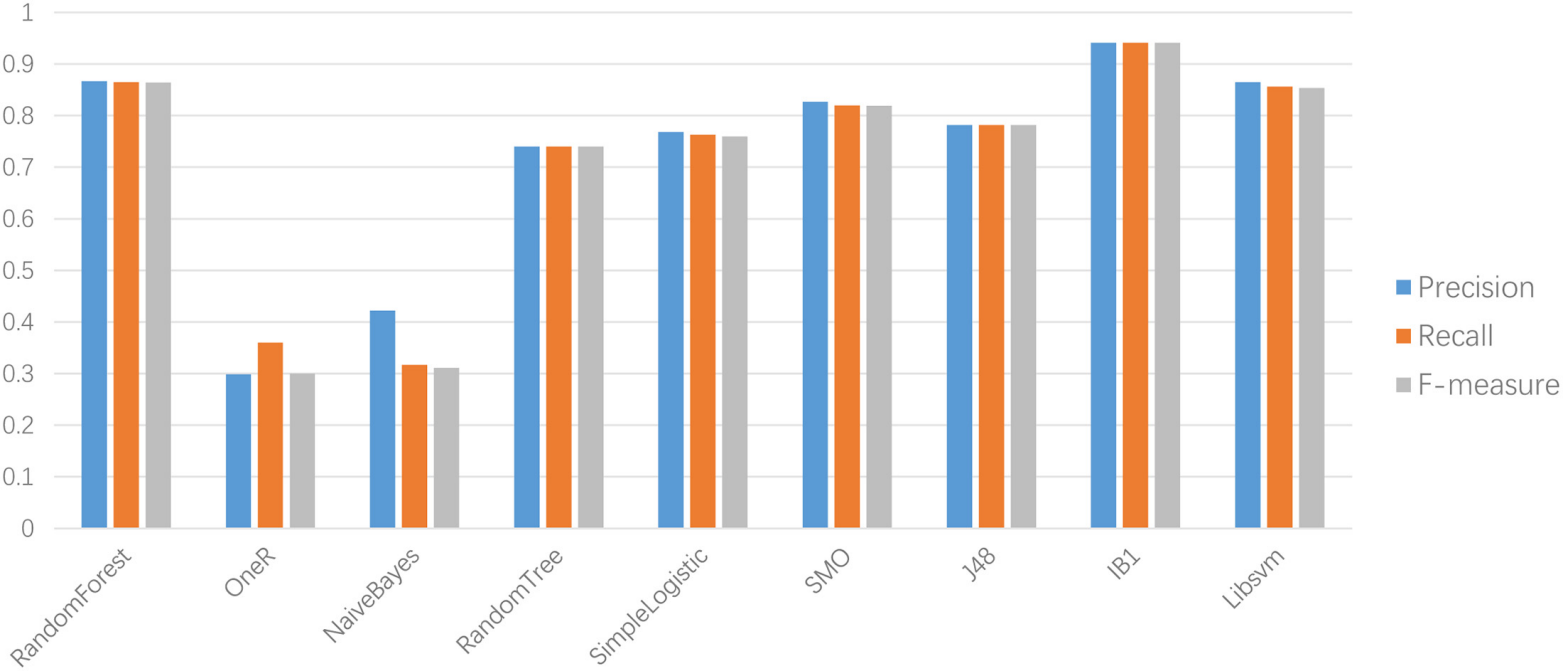
Results of ACC method on different classifiers.

First, we evaluated the importance of distance parameter *λ* in the ACC feature representation algorithm; 94.1% accuracy is attained for the dataset with similarity below 65% when *λ* is set to 1. With the increase in parameter *λ*, the improvement is not evident (only 0.1% increase), but time consumption is multiplied. This condition implies that the homology among adjacent amino acids is high. Second, we compared the performance of ACC method in different classifiers. IB1, which was built by KNN where neighbor k was set to 1, yielded the best results. The comparison results are shown in Fig 2.

**Fig 3:**
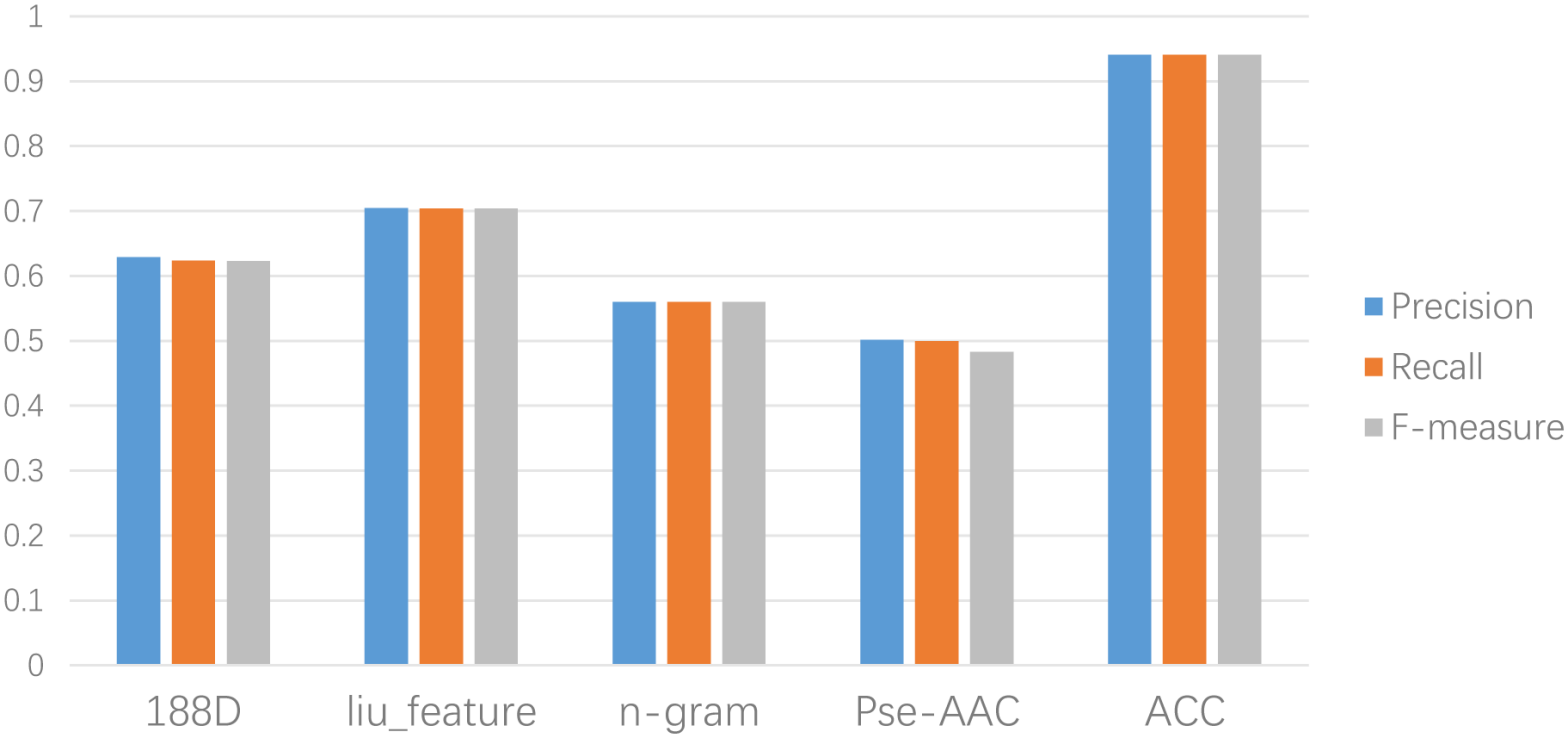
Results of fivefeaturerepresentationmethods on IB1 classifier.

We also compared ACC with other popular protein prediction methods, such as 188D[24] (which considers the constitution, physicochemical properties[25], and distribution of amino acids), liu_feature (820D)[26] (which combines evolution information extracted from frequency profiles with sequence-based kernels for protein remote homology detection), n-gram (20D)[27] proposed by Browm et al. (which denotes the feature vectors by probability calculation), Pse-AAC (420D) originally proposed by Chou[28, 29] (which has been comprehensively applied for diverse biological sequence analyses as an effective protein descriptor[30–38], and DNA descriptor[39–42]. As shown in Fig 3, the advantage of the ACC algorithm is obvious.

Aside from these five feature representation methods, we also tested two other enzyme-oriented online platforms. The first one is EzyPred. We randomly extracted 10 enzyme sequences from each class within one multifunctional enzyme as the test dataset and obtained 80% accuracy, which is lower than the 93.7% accuracy mentioned in the paper. The public test website http://www.csbio.sjtu.edu.cn/bioinf/EzyPred/EzyPred is free to the public. The second platform is EFICAz2.5[11, 43]. We obtained 86.4% accuracy with the code obtained from the link http://cssb.biology.gatech.edu/skolnick/webservice/EFICAz2/index.html. This accuracy value is lower than the 92% accuracy mentioned in the paper.

### Multifunctional enzyme classification

We applied the ACC method to multifunctional enzyme classification according to the results of monofunctional enzyme prediction. Given that KNN works well in monofunctional enzyme classification, we focused on classifiers (IBLR_ML[44]/MLkNN[45]/BRkNN[46]) whose kernel is the KNN algorithm with the aid of MULAN. Two other classifiers (RakEL[47]/HOMER) were also tested. From Table 3, we can see that the classifier IBLR_ML obtained the best average precision of 95.54%. Classifiers MLkNN and BRkNN also produced good results.

**Table 3:** Cross-validation results of Multi-Label classifiers.

	IBLR_ML	MLkNN	BRkNN	RAkEL	HOMER
**Micro-averaged Precision**	0.9239	0.9202	0.9251	0.9117	0.9070
**Micro-averaged Recall**	0.9128	0.919	0.9159	0.9117	0.8869
**Micro-averaged F-Measure**	0.9183	0.9196	0.9205	0.8628	0.8968
**Macro-averaged Precision**	0.9176	0.9134	0.9189	0.9181	0.9006
**Macro-averaged Recall**	0.9021	0.9103	0.907	0.8039	0.8759
**Macro-averaged F-Measure**	0.9097	0.9118	0.9128	0.8559	0.8879
**Average Precision**	0.9554	0.9542	0.9442	0.9267	0.9305

To test the classification performance of the multifunctional enzyme further, we performed cross validation on the multifunctional enzyme only. To ensure data reliability and experimental accuracy, the threshold of data redundancy was set to 0.9. Then, we obtained the dataset in Table 4. Table 5 shows that 89.4% average precision was obtained.

**Table 4:** Distribution of multifunctional enzyme after de-redundance (0.9).

EC 1	EC 2	EC 3	EC 4	EC 5	EC 6	Total
861	994	1426	927	290	91	4589

**Table 5:** Cross-validation results of Multi-Label classification on multifunctional enzymes only.

	IBLR_ML	MLkNN	BRkNN	RAkEL	HOMER
**Micro-averaged Precision**	0.8406	0.8374	0.8279	0.8090	0.7519
**Micro-averaged Recall**	0.8178	0.8209	0.8285	0.8126	0.8233
**Micro-averaged F-Measure**	0.8290	0.8290	0.8282	0.8108	0.7859
**Macro-averaged Precision**	0.6792	0.6746	0.7341	0.7364	0.6056
**Macro-averaged Recall**	0.6705	0.6761	0.7379	0.6917	0.6619
**Macro-averaged F-Measure**	0.6737	0.6747	0.7347	0.7004	0.6305
**Average Precision**	0.8940	0.8930	0.8583	0.8910	0.8407

To obtain good results, the five classifiers shown in Table 5 are combined into one. Precision increased to 91.25% with the TOP3 combination rule.

In statistical prediction, the independent dataset test, subsampling or K-fold crossover test and jackknife test are the three cross-validation methods often used to check a predictor for its accuracy[48]. However, among the three test methods, the jackknife test is deemed the least arbitrary that can always yield a unique result for a given benchmark dataset[49]. Accordingly, the jackknife test has been increasingly used and widely recognized by investigators to examine the quality of various predictors[31, 32, 34, 39, 40, 50–54]. However, for saving computational time, the 5-fold cross-validation was used in this study.

## Conclusion

We have explored a new method of multifunctional enzyme prediction. Considering the position relation and homology among amino acids[55], we extracted sequence features by using ACC method and performed prediction by using the KNN algorithm. The cross-validation test results indicate that our method outperforms other existing algorithms in datasets with similarity below 65%. Accuracy values of 94.1% in monofunctional enzyme classification and 95.54% in multifunctional enzyme classification were achieved. Compared with other existing prediction methods in the field of multifunctional enzyme class prediction, our method demonstrates better versatility and effectiveness. A public prediction—recognition platform is provided at http://server.malab.cn/MEC/. Our work is expected to be helpful for enzyme prediction in the future.

Our work just focused on the features and multi-label classifier. Some other machine learning techniques, such as feature selection[56], training sample selection[57, 58], ensemble learning[59–61], network features[62–64], imbalance classification[65, 66], ought to be considered in the next step. It is worth noting that there are many other potential tools for enzyme prediction, such as, evolutionary computation[67, 68] and spiking neural models[69–76]. Furthermore, parallel techniques, such as Map Reduce[77, 78], should also be considered for big testing data in the future.
